# Structural Design and Physical Mechanism of Axial and Radial Sandwich Resonators with Piezoelectric Ceramics: A Review

**DOI:** 10.3390/s21041112

**Published:** 2021-02-05

**Authors:** Wenjie Wang, Yi Jiang, Peter J. Thomas

**Affiliations:** 1 School of Aerospace Engineering, Beijing Institute of Technology, Beijing 100081, China; jyjybit@163.com; 2 School of Engineering, University of Warwick, Coventry CV4 7AL, UK; P.J.Thomas@warwick.ac.uk

**Keywords:** piezoelectric resonators, piezoelectric ceramics, piezoelectric effect, sandwich structure, structural evolution, energy conversion

## Abstract

Piezoelectric ceramics are inexpensive functional materials which are widely used in sonar detection, home appliances, meteorological detection, telemetry and environmental protection and other applications. Sensors fabricated from these materials are compact and have fast response characteristics. Their underlying functional methodology is based on the direct piezoelectric effect whereby very small mechanical vibration signals are converted into electrical signals. Piezoelectric resonators are based on the reverse piezoelectric effect and they are widely used for the control of precision instruments and precision machinery, microelectronic components, bioengineering devices and other in applications requiring components to provide precision control of the relevant functional mechanism. In this paper, the structural evolution and design mechanism of sandwich resonators based on piezoelectric materials are reviewed, and the advantages and disadvantages of different structures are compared and analyzed. The goal is to provide a comprehensive reference for the selection, application and promotion of piezoelectric resonators and for future structural innovation and mechanism research relevant to sandwich resonators.

## 1. Introduction

Piezoelectric ceramics are functional ceramic material exploiting the piezoelectric effect. They enable conversion between mechanical and electrical energy. The difference between piezoelectric functional ceramics and traditional piezoelectric quartz crystal materials [1,2,3,4] mainly lies in the crystal phase of the main components. The traditional typical piezoelectric quartz crystal does not contain ferroelectric components while all piezoelectric functional materials have ferroelectric grains. Ceramic materials are polycrystalline aggregates with randomly oriented grains and, therefore, the spontaneous polarization vector of each ferroelectric grain in piezoelectric ceramic materials is also disorderly oriented. Such a state of disordered orientation cannot display macroscopic piezoelectric characteristics. The modification of the microscopic intrinsic random orientation of piezoelectric functional materials is an important issue affecting the overall macroscopic piezoelectric properties. Once a piezoelectric functional ceramic has been produced, the end face of the material is treated by a composite electrode and the external strong dc electric field is used for polarization treatment. That is, under the action of an external electric field, the polarization vectors of the original random orientation are preferentially aligned along the direction of the electric field. After eliminating the external strong dc electric field, the polarized piezoelectric ceramic material will retain part of the macro residual polarization strength, so that the ceramic material has certain piezoelectric characteristics [5,6].

The piezoelectric effect was first discovered by Pierre Curie and Jacques Curie [7,8,9] for tourmaline in 1880. Subsequently they experimentally verified the existence of the reverse piezoelectric effect and determined the direct and reverse piezoelectric effect constants. Voigt [10] found the piezoelectric effect of dielectric materials. Langevin [11,12,13,14,15,16] used quartz materials to develop underwater ultrasonic detectors for the detection of enemy submarines. In 1920, Valasek [17,18,19,20] proposed the concept of “ferroelectrics” after Rochelle salt was discovered. The first piezoelectric ceramic devices were Barium-titanate pickups [21] and these were developed in 1947. Piezoelectric ceramics are classified into four categories [22] according to their crystal structure: perovskite structure, tungsten-bronze structure, bismuth layer structure and pyrochlore structure. However, there also exists and alternative classification scheme [23] based on different basic components. They are unit system ceramics, binary system ceramics [24,25,26,27], ternary system ceramics [28,29,30,31,32], quaternary system ceramics [33,34,35,36] and other system ceramics.

At present, most tag names of the piezoelectric ceramics on the market are based on different Barium titanate components and PZT4i-PZT8i (or P4i-P8i, i = 1, 2, 3 …) are recognized as the mainstream names due to their different applications. P4 is used to launch or receive signals. P5 is mainly used for drive and detection. P6 represents high stability. P7 stands for high frequency and Lead Zirconate Titanate. P8 is always used for high-power applications. The existing different ceramic types, together with typical applications and their characteristics [37] are summarized in Table 1.

In addition to the dielectric and elastic properties of common dielectric materials, piezoelectric ceramics also have piezoelectric properties. After polarization treatment, piezoelectric ceramics display anisotropy such that the values of each performance parameter differ in different directions. It makes the number of performance parameters of piezoelectric ceramics much more than the general isotropic dielectric ceramics. The performance parameters of piezoelectric ceramics are the important basis for its wide application, such as elastic compliant constant, dielectric constant, dielectric loss, quality factor, electromechanical coupling coefficient and piezoelectric constants, etc. These isotropic and anisotropic performance parameters of different piezoelectric ceramics [37,38] are summarized in Table 2. However, there may exist slight difference between the reference values of Table 2 and the actual values encountered in practice due to different production formulas being used in different factories. Manufacturers provide data sheet of their particular calibrated values.

## 2. Physical Mechanism and Application of Piezoelectric Effect

Piezoelectric properties give rise to the direct and the inverse piezoelectric effect. The direct piezoelectric effect can change mechanical energy into electric energy, which can be exploited for piezoelectric sensors, while the inverse piezoelectric effect is used for piezoelectric actuators because electric energy is converted into mechanical energy.

**Figure 1:**
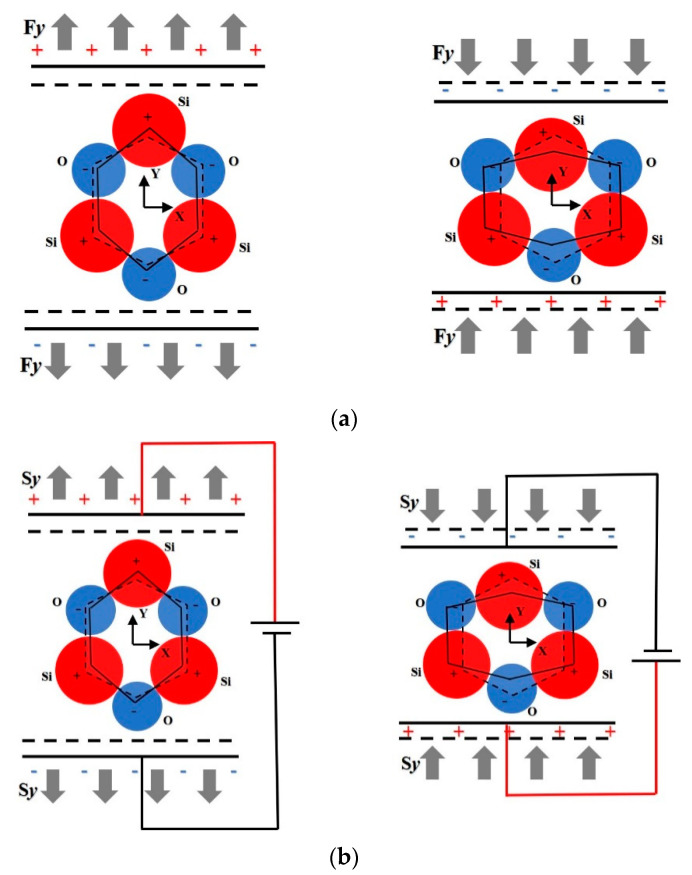
Schematic diagram illustrating the piezoelectric effect. (**a**) Direct piezoelectric effect, (**b**) Inverse piezoelectric effect.

The piezoelectric effect arises from the molecular structure of quartz [39] as illustrated in Figure 1. In the figure red circles represent *Si* (positive charges) atoms and blue circles represent oxygen atoms *O* (negative charges). The dotted line displays the original symmetric atom constellation existing when no mechanical forces act on the material and the centres of mass for the positive and negative charges are at the original position. When tensile or compressive stresses, *Fy*, act on the structure along its electrical axis *Y* structural deformations occur. This gives rise to the charge distribution illustrated in in Figure 1a. The centres of mass of the positive and negative charges split and a dipole moment is created. Therefore, the charges can be measured on two opposing surfaces due to this redistribution. That is the direct piezoelectric effect. The inverse piezoelectric effect arises as a reverse process of the direct piezoelectric effect. When an external electric field is applied to a piezoelectric material, the material itself will undergo mechanical deformation. Similarly, the dipole moment and displacement direction *Sy* are oriented in an opposite direction when different external electric fields are applied, as illustrated in Figure 1b.

The deformation of the piezoelectric material is minute, it usually does not exceed one thousandth of its own size. Due to such small deformation, piezoelectric actuators can be widely used in the control of precision instruments [40] and precision machinery [41], microelectronics technology [42], bioengineering [43] and other precision fields [44] to enable precise control of the relevant mechanism. Piezoelectric ceramics are used as frequency control devices such as resonators and filters, they have been widely used in communication systems [45] and they are gradually replacing traditional electromagnetic equipment. Piezoelectric materials can improve the anti-interference of multi-channel communication equipment and have the characteristics of high precision, good frequency stability and wide-band application. For practical applications the required components are typically small and light, the materials are not easy to be damped and have a long service life benefitting mass and cost reduction.

**Figure 2:**
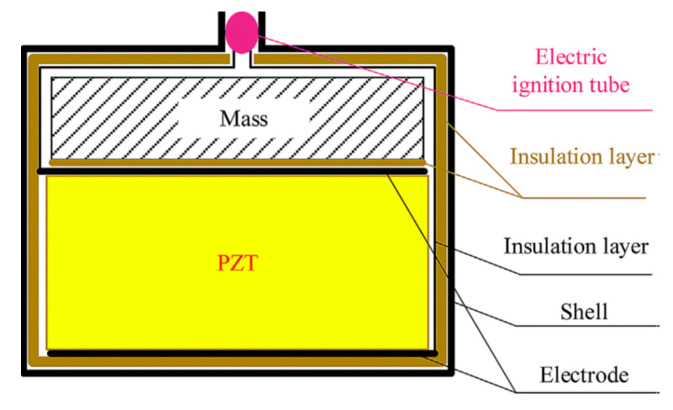
Structure of typical self-powered overload ignition device.

Piezoelectric ceramics can be used to manufacture piezoelectric igniters [46,47] (cf. Figure 2), mobile X-ray power supplies [48] or shell detonating devices [49]. A gas electronic lighter with two thin piezoelectric ceramic columns, instead of an ordinary flint, can continuously ignite a million times. Similarly, a buzzer made of piezoelectric ceramics enables alarms or toys to emit different sounds [50,51,52]. Solvay [53], for example, manufactures sensors that resemble capacitors in loudspeakers or buzzers, which on the application of an external force result in the development of an electromagnetic field. Piezoelectric ceramics transforming electric energy into ultrasonic vibration can be exploited to explore the location of fish, enable ultrasonic cleaning, non-destructive testing of metal and ultrasonic medical treatment. It can also be used in the context of various ultrasonic cutters, welding devices and soldering irons to process plastic and metal.

Piezoelectric ceramics are sensitive to external forces and convert extremely weak mechanical vibration into electrical signals. With this characteristic, piezoelectric ceramics are widely used in sonar systems [54]. These can also detect fish groups or explore seabed topography, etc. In the military field, all submarines are equipped with sonar systems referred to as “underwater scouts”. These devices are indispensable equipment for underwater navigation, communication, reconnaissance of enemy ships, cleaning up enemy mines, and a powerful tool for the development of marine resources. Sonar systems contain a core component that is the piezoelectric ceramic underwater acoustic transducer. When the acoustic signal emitted by the piezoelectric transducer arrives at an underwater target, it produces a reflection which is received by another receiving underwater acoustic transducer enabling target location. Currently piezoelectric ceramic remains to be one of the best materials for manufacturing underwater acoustic transducer [55,56,57,58]. Another application is the piezoelectric ceramic sensor [59]. This is used to measure the change of chamber pressure and the pressure of the muzzle shock wave at the moment when a bullet is fired. They can measure both high pressure and low pressure. AAC Technologies [60] manufactures integrated micro-component solutions, which provide acoustic and non-acoustic components. The two types of piezoelectric ceramics solutions, piezo actuators and touch sensors, are used in cell phones for feedback through vibrations and are advantageous due to their low power consumption and quick response ability.

**Figure 3:**
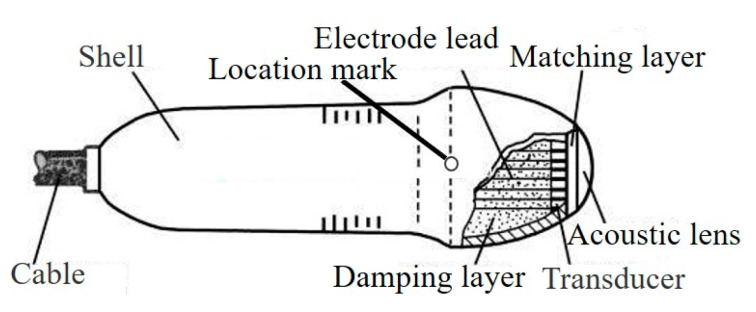
Medical ultrasonic transducer.

Doctors use piezoelectric ceramic probes [61], such as that shown in Figure 3, to examine parts of the human body. To this end ultrasonic waves are emitted and sent to the tissue of the human body to generate an echo. The echo is detected and displayed on a fluorescent screen, so that doctors can understand the internal condition of the human body.

**Figure 4:**
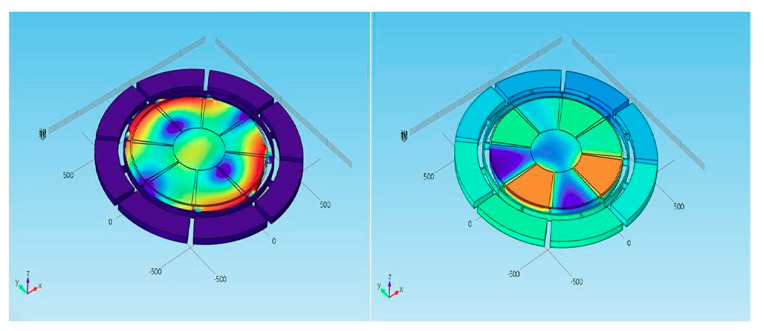
Piezoelectric gyroscope.

Piezoelectric gyroscopes [62,63,64,65] fabricated from piezoelectric ceramics, illustrated in Figure 4, are attached to the rudder of spacecraft and artificial satellites, and thereby guarantee a stable fixed route. Traditional mechanical gyroscopes, with short life, poor accuracy, and low sensitivity, cannot meet the requirements of modern spacecraft and satellite system. However, piezoelectric gyroscopes are sufficiently small and have high sensitivity and reliability. CEDRAT Technologies [66] produces such piezoelectric smart materials under the brand name APA, SPEED SENSOR, PPA, APA400M-MD, CAu10, CAu20, SPC45, APA60SM and others, which can be found in airplanes, helicopters, missiles, military vehicles, Micro aerial vehicles (MAV), satellites and nano satellites, spacecraft, Unmanned aerial vehicles (UAV).

**Figure 5:**
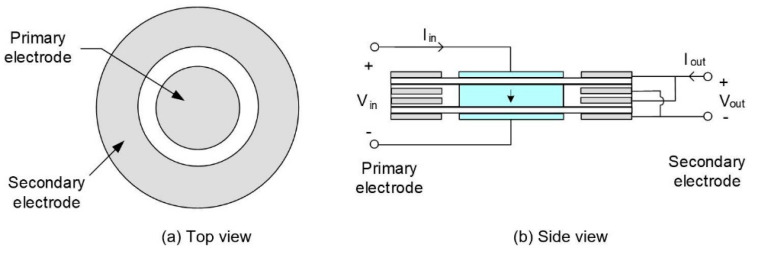
Structure of the radial vibration mode disk piezoelectric transformer.

Geological detectors with piezoelectric ceramic elements [67,68] are used to judge the geological conditions of the strata and explore underground mineral deposits. Piezoelectric ceramic sensors play an important role in the measurement of detonations caused by the mismatch of combustion pressure, vacuum, and ignition angle in automobile engine. Another common application of piezoelectric ceramics is the type of ceramic transformers illustrated in Figure 5 [69,70,71], which is smaller in volume and lighter in weight than traditional instruments. Their efficiency can reach 60~80%. They can withstand high voltages of 30,000 volts and keep the voltage stable. Arkema [72] developed a range of ultra-high added value electroactive fluorinated polymers.

In addition to the companies referred to above, there are many other famous companies [73,74] producing piezoelectric ceramics and related products in the world. For example, Kyocera [75] developed an innovative piezoelectric actuator audio “Smart Sonic Sound”, which was utilized in a flat-screen television for the first time by LG. Vesper Technologies [76], with origins at the University of Michigan, has a major leap over the capacitive MEMS microphones that have dominated the market for over a decade. Cambridge Touch Technologies (CTT) [77], from the Centre for Advance Photonics and Electronics (CAPE) of Cambridge University, has developed a next generation 3D touch technology enabling mobile devices to sense both the location and force of multi-touch inputs. This improves on the first generation of 3D technologies and is more scalable and cost-effective without any decrease in battery life.

In summary, resonators made of piezoelectric ceramics are ubiquitous in a wide range of applications across many different fields. It is no exaggeration to say that it is everywhere and indispensable in our daily life. Therefore, it is crucial to be familiar with the underlying principles of their operation and their mechanical structure, whether it is for high-end equipment or devices of daily use.

## 3. Theoretical Mechanism of Numerical Simulation

The typical sandwich structure in a cymbal transducer usually includes two endcaps and one piece of piezoelectric ceramic. The core component may be one of the three structures. That is a piezoelectric ceramic sheet, a piezoelectric ceramic sheet with a metal ring or a piezoelectric ceramic ring. In this section the theoretical mechanisms and electromechanical equivalent principles of these common structures will be introduced separately.

### 3.1. Piezoelectric Ceramic Sheet

**Figure 6:**
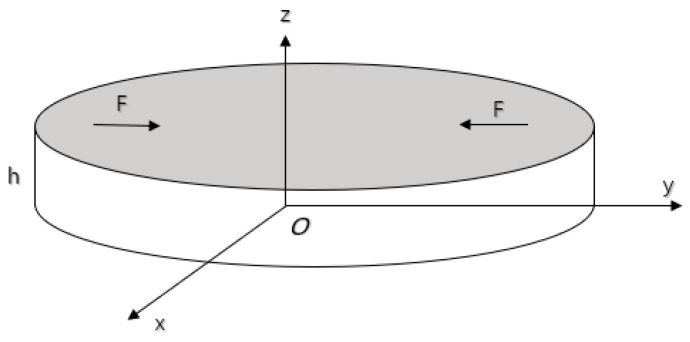
Schematic diagram of piezoelectric ceramic disc.

The polarization direction of the piezoelectric disk and the direction of the excitation of the electric field are assumed to be orientated along the axis of the disk (z axis of the coordinate system in Figure 6). In Figure 6, the nomenclature for the components of piezoelectric ceramic disc is as follows: disc thickness *h*, disc diameter *a* and radial force *F*.

**Figure 7:**
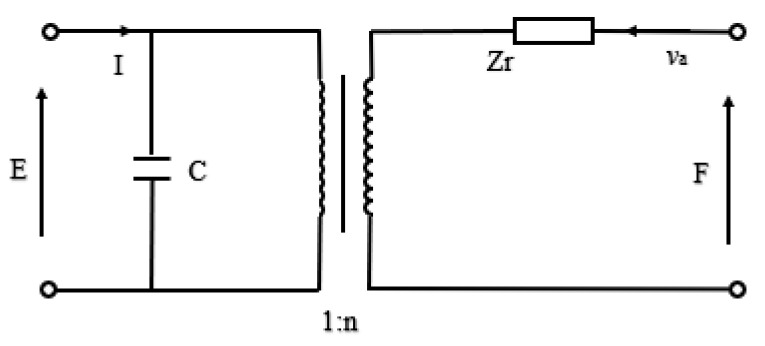
Electromechanical equivalent diagram of radial vibration of piezoelectric ceramic disk.

For the design and analysis of a transducer, the equivalent circuit based on Kirchhoff’s law [78] is a simple and intuitive effective analysis method. The expressions for radial vibrations of the disk, equations of state of the piezoelectric medium and boundary conditions can be found in the reference [79,80,81]. The combination of equations enables to represent the oscillating resonator in the form of an equivalent circuit (Figure 7).

The values of the quantities in Figure 7 were described in detail by the formulas in References [80,81]. Then, the relationships in Figure 7 can be expressed as,

(1) $$F=Z_{r}v_{a}+nE,\;n=\frac{2\pi ad_{31}}{s_{11}^{E}\left(1-v_{12}\right)}$$

(2) $$Z_{r}=j\cdot Z_{a}\left[\frac{1-v_{12}}{ka}-\frac{J_{0}\left(ka\right)}{J_{1}\left(ka\right)}\right],\;Z_{a}=\rho ES_{a}$$

(3) $$I=j\omega CE-nv_{a},\;C=\frac{\pi a^{2}\varepsilon }{h}\left[1-\frac{2d_{31}^{2}}{\varepsilon \left(s_{11}^{E}+s_{21}^{E}\right)}\right]$$

where, *Z_r_* is the mechanical impedance and *Z_a_* is the specific mechanical impedance, *S_a_* is the area of outer surface, ρ is the density, *v_a_* is the radial vibration velocity, *E* is the terminal voltage of piezoelectric ceramic disk, *k* is the wave number of radial vibration, *J_i_* represents Bessel functions and *d*_31_ is the piezoelectric strain constant. The quantity *n* represents the electromechanical conversion coefficient of radial vibration, *v*_12_ is the Poisson coefficient of piezoelectric material, *I* is the current, *C* is the capacitance, *ε* is the dielectric constant, *ω* is the angular frequency,$\;s_{ij}^{E}$ is the elastic compliance constant under constant electric field strength.

According to the admittance equation, the frequency expressions in Figure 7 can be obtained. When the conductance is close to zero, the frequency equation in the anti-resonance state can be expressed as

(4) $$1-k_{p}^{2}+\frac{k_{p}^{2}\left(1+v_{12}\right)J_{1}\left(ka\right)}{kaJ_{0}\left(ka\right)-\left(1-v_{12}\right)J_{1}\left(ka\right)}=0$$

where, *k_p_* is the electromechanical coupling coefficient.

When the conductance is close to infinity, the frequency equation in the resonance state can be expressed as

(5) $$kaJ_{0}\left(ka\right)-\left(1-v_{12}\right)J_{1}\left(ka\right)=0$$

### 3.2. Piezoelectric Ceramic Disc with a Metal Ring

**Figure 8:**
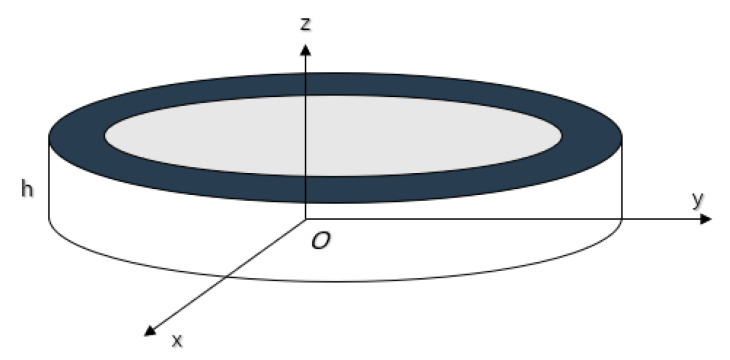
Schematic diagram of PZT with metal ring.

In order to improve the strength and pressure resistance of piezoelectric transducers, the piezoelectric ceramic sheet is embedded into a metal ring by means of thermal expansion and cold contraction, as shown in Figure 8. The vibration characteristics and electromechanical characteristics of piezoelectric ceramic sheets are the same as those in the Section 3.1.

The radial stress [80,81] is expressed as,

(6) $$F_{r2}=\left(Z_{1m}+Z_{3m}\right)v_{r2}+Z_{3m}v_{r3}$$

(7) $$F_{r3}=\left(Z_{2m}+Z_{3m}\right)v_{r3}+Z_{3m}v_{r2}$$

where, *F_r_*_2_ and *v_r_*_2_ are the force and velocity on the piezoelectric disk, *F_r_*_3_ and *v_r_*_3_ are the force and velocity on the metal ring, *Z_im_* (*i* = 1, 2, 3) is equivalent mechanical impedance of metal ring.

**Figure 9:**
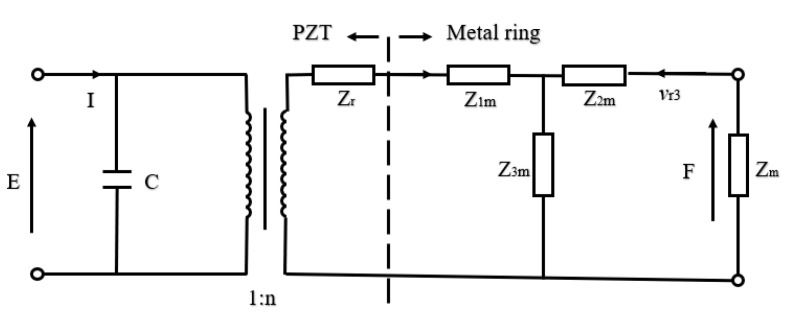
Electromechanical equivalent circuit of PZT with metal ring.

The combination of the metal ring and the piezoelectric ceramic is shown in Figure 8. In practical application, the piezoelectric ceramic disc is rigidly connected with the metal ring and the metal end cap. Due to the bending and tension of the end cap and the complexity of the shape of the end cap itself, it is difficult to obtain the exact analytical solution of the whole transducer. However, according to the rigid contact between the inner side of the metal ring and the outer cylinder of the piezoelectric ceramic disk, it has the continuous conditions of continuous vibration speed and continuous force. Assuming that the impedance *Z_M_* of the metal end cap is known, the electromechanical equivalent circuit diagram of the piezoelectric material transducer can be drawn, as shown in Figure 9.

From the electromechanical equivalent circuit diagram in Figure 9, the electromechanical equation of the piezoelectric transducer and the electric admittance relation can be obtained successively [80,81].

(8) $$Y=\frac{I}{E}=G+jB=j\omega C+\frac{n^{2}}{Z_{r}+Z_{1m}+\frac{Z_{3m}\left(Z_{2m}+Z_{m}\right)}{Z_{m}+Z_{2m}+Z_{3m}}}$$

where, *C* is direct capacitance, *Z_r_* and *Z_m_* are the impendence of PZT and metal ring.

The resonance frequency and anti-resonance frequency can be obtained from the admittance equation.

### 3.3. Piezoelectric Ceramic Ring with a Metal Ring

**Figure 10:**
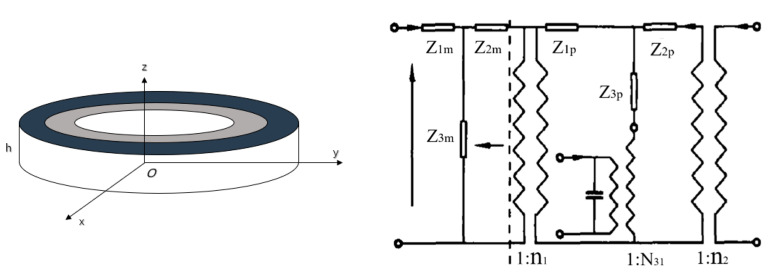
Schematic diagram of piezoelectric ring with metal ring (**left**) and its electromechanical equivalent circuit (**right**).

The radial composite ultrasonic transducer is composed of a piezoelectric ceramic ring and a metal ring [82,83,84]. In Figure 10, the inner and outer radius and thickness of the metal ring are *R*_1_, *R*_2_ and *h* respectively. The inner and outer radius and thickness of the piezoelectric ceramic ring, polarized in the thickness direction, are *R*_2_, *R*_3_ and *h* respectively. Under the external electric field excitation, the transducer can produce two vibration modes. When the radius of transducer is much larger than the thickness, the resonance frequency of the radial vibration is much smaller than that of thickness vibration.

In Figure 10, according to the same principle, the radial force can be obtained [79].

(9) $$F_{r1}=\left(Z_{1m}+Z_{3m}\right)v_{r1}+Z_{3m}v_{r2}$$

(10) $$F_{r2}=\left(Z_{2m}+Z_{3m}\right)v_{r1}+Z_{3m}v_{r1}$$

The input impedance *Z* is

(11) $$Z=\frac{E}{I}=\frac{Z_{m}}{N_{31}^{2}+j\omega C_{or}Z_{m}}$$

where, *N*_31_ is electromechanical conversion coefficient.

The input impedance is pure reactance without considering internal loss and load impedance. Then, the resonance frequency and anti-resonance frequency can be obtained from Equation (11).

### 3.4. Cascaded Piezoelectric Transducer

**Figure 11:**
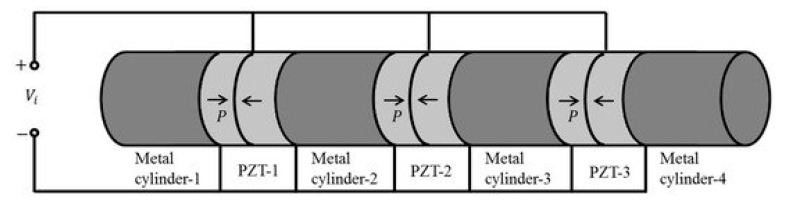
Cascaded piezoelectric transducer.

Figure 11 illustrates a cascaded piezoelectric transducer in series with four metal columns and three sets of piezoelectric ceramic plates [85,86]. Here *P_i_* is the polarization direction, *L_i_* is the length of metal column and *p_i_*, *l_i_* are the number and length of piezoelectric ceramic.

When mechanical and dielectric losses are ignored and when the longitudinal size of the cascaded transducer is much larger than the transverse size, the electromechanical equivalent circuit of Figure 10 is obtained based on one-dimensional theory.

**Figure 12:**
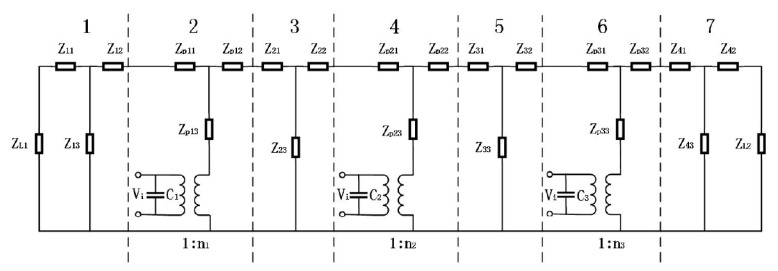
Electromechanical equivalent circuit of cascaded piezoelectric transducer.

In Figure 12, *Z_Li_* is the mechanical impedance, *V_i_* is the input voltage, *C_i_* is direct capacitance, *n_i_* is the electromechanical conversion coefficient. Then [86],

(12) $$C_{i}=\left[p_{i}\varepsilon_{33}^{T}\left(1-K_{33}^{2}\right)S\right]/l_{i}$$

(13) $$n_{i}=d_{33}S/S_{33}^{E}l_{i}$$

(14) $$Z_{j1}=Z_{j2}=jZ_{j}tan(k_{j}L_{j}/2)$$

(15) $$Z_{j3}=Z_{j}/\left[jsin(k_{j}L_{j})\right]$$

(16) $$Z_{pi1}=Z_{pi2}=jZ_{0}tan(p_{i}k_{0}L_{i}/2)$$

(17) $$Z_{pi3}=Z_{0}/\left[jsin(p_{i}k_{0}l_{i})\right]$$

(18) $$Z_{e}=Z_{e1}Z_{e2}Z_{e3}/\left(Z_{e1}+Z_{e2}+Z_{e3}\right)$$

where, *Z_ei_* (*i* = 1, 2, 3) is the input impedance. $\varepsilon_{33}^{T}$, *k*_33_, *d*_33_ and $S_{33}^{E}$ are the dielectric constant, the piezoelectric constant, the electromechanical coupling coefficient, and the elastic compliance constant of the piezoelectric material.

According to the same method, when the total input impedance is zero or infinite, the resonance frequency *f_r_* and anti-resonance frequency *f_a_* can be obtained from Equation (18). The effective electromechanical coupling coefficient *K_effc_* and mechanical quality factor *Q_m_* can be expressed as

(19) $$K_{effc}={\left[1-{\left(f_{r}/f_{a}\right)}^{2}\right]}^{1/2}$$

(20) $$Q_{m}=f_{a}{\left(Z_{a}/Z_{r}\right)}^{1/2}/\left[2(f_{a}-f_{r})\right]$$

## 4. Different Sandwich Structures of Piezoelectric Resonator

**Figure 13:**
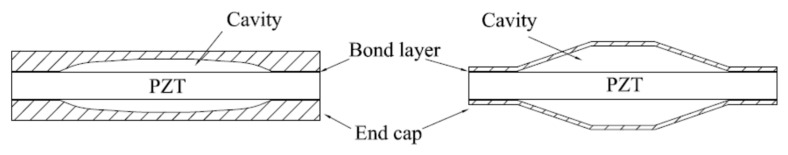
Structure diagrams of Moonie (**left**) and Cymbal (**right**).

Recently a new breakthrough in the application of nanotechnology has led to benefits for the manufacturing process of piezoelectric materials. Worldwide lead-free piezoelectric ceramics are now being developed vigorously to protect the environment and pursue health. Application of piezoelectric ceramic materials in intelligent structures began in the late 1980s. Scientists from the Pennsylvania State University developed a V-shaped bent and tensioned cymbal intelligent structure, which is referred to as “moonie” [87,88,89] and “cymbal” [90,91,92,93] because its shape is similar to the crescent moon. The structures are shown in Figure 13. The two intelligent structures are similar, both of which are a piezoelectric ceramic sheet (PZT) sandwiched between two metal end caps. The structure can effectively transform the small radial vibration of a piezoelectric ceramic sheet into large axial movement of the metal end cap resulting in the output efficiency being greatly improved. Patents for these innovations have been applied for.

In early cymbal intelligent structures, piezoelectric ceramic sheets (PZT) are usually connected with the metal end caps on both sides by a bonding layer. The adhesives, such as ethyl α-cyanoacrylate, modified acrylate and epoxy resin, have strength problems and, therefore, they cannot be used in deep water or in a high-pressure environment. However, the thickness of the bond layer is bound to have an impact on the performance of the cymbal. In most studies, the connection between PZT and the metal end cap is simplified as a spring mass damping system (SMD) [94,95]. When the thickness of the bond layer is not considered, it will lead to a large error of 12.4% [96]. Wu et al. [97] found that the different bond strength of different binder materials results in a change of impedance after bond between adhesive and PZT.

**Figure 14:**
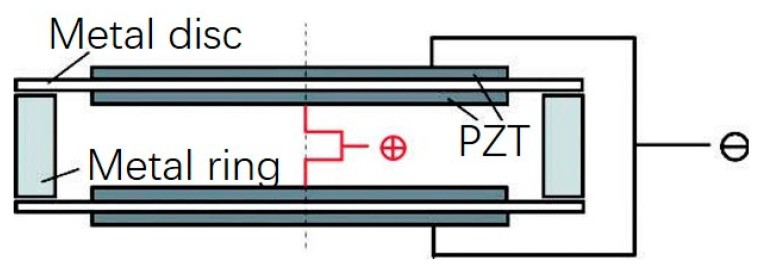
Structural diagram of the double-sided triad-curved hydrophone.

In order to realize the high sensitivity of low frequency acoustic waves, the double-sided triad-curved hydrophone shown in Figure 14 is simulated and optimized by means of the COMSOL finite element software [98]. The maximum size of the hydrophone prototype is 45 mm, and the maximum receiving sensitivity level is −178 dB in the frequency range of 0.5–2.5 kHz.

**Figure 15:**
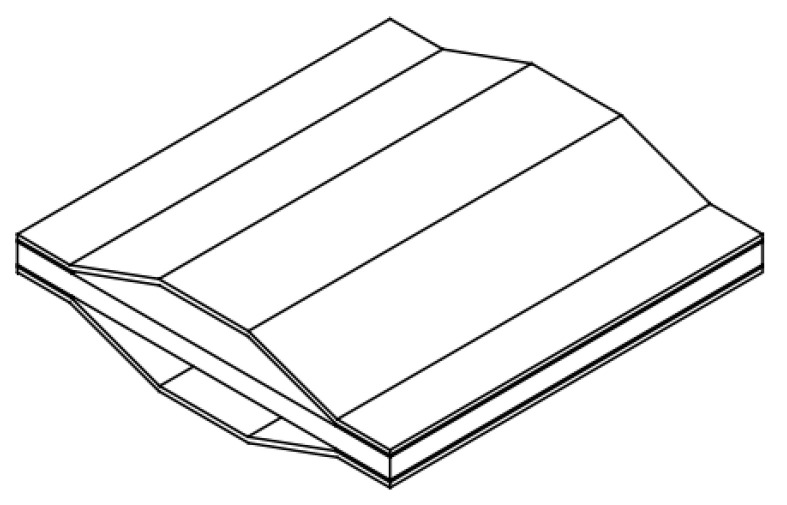
A derivative cymbal transducer with rectangular endcap.

A derivative cymbal transducer in Figure 15 has been designed from rectangular single crystal material and the whole structure is a sandwich structure with rectangular metal end caps [99]. This structure can make full use of the optimal crystal plane direction of single crystal material. Results of a finite element analysis show that the cymbal transducer has good displacement characteristics, and the hysteresis effect can be reduced by selecting the appropriate crystal orientation.

**Figure 16:**
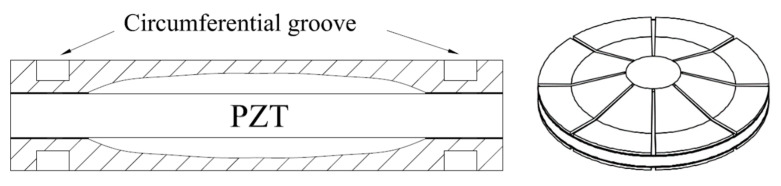
Structure diagrams of cymbal with circumferential groove (**left**) and radial groove (**right**).

Due to the stress concentration at the inner cavity edge of the bonding layer, the efficiency of the intelligent structure is relatively low [100,101]. In order to solve a series of problems in the adhesion of the boundary layer, some scholars have slotted the metal end cap with circular groove and radial groove, which are shown in Figure 16. Sugawara [102] proposed that the circular groove structure of metal end cap cannot completely eliminate the stress concentration, but a further stress concentration on the endcaps will result in fatigue damage. In addition, the annular groove will also increase the complexity of the manufacturing process and the manufacturing cost.

A different type of end cap with radial grooves was proposed by Ke et al. [103,104], which effectively solves the problem of circular stress concentrations and also improves the energy conversion coefficient. Following the optimization of the number of slots, the end cap meets the engineering needs of different fields. However, this type of radial groove structure is not suitable to resist conditions of high pressure and it can therefore not be used in applications involving underwater intelligent materials.

**Figure 17:**
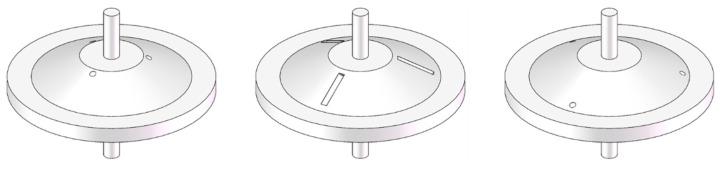
The cymbal transducer with pressure compensation.

The cymbal transducer with pressure compensation [105] in Figure 17 is perforated on the cymbal end cap to connect the cavity of the end cap with the external liquid. This structure not only achieve the pressure balance of the cavity, but also improves the static pressure resistance performance while the static pressure sensitivity is not affected. There are three perforation schemes. The first involves three uniformly distributed circular holes near the top of the cone of the conical rotating surface of the end cap. The second has three uniformly distributed slot holes on the conical rotating surface of the end cap. The third features three uniformly distributed circular holes near the bottom of the cone. The experimental results show that the performance of the last two schemes is better than that of the first scheme.

**Figure 18:**
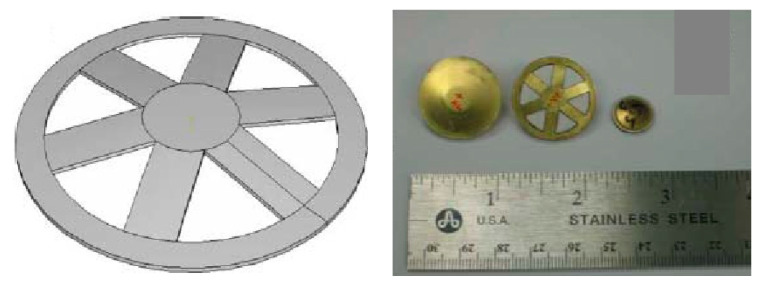
Hub cymbal transducer.

**Figure 19:**
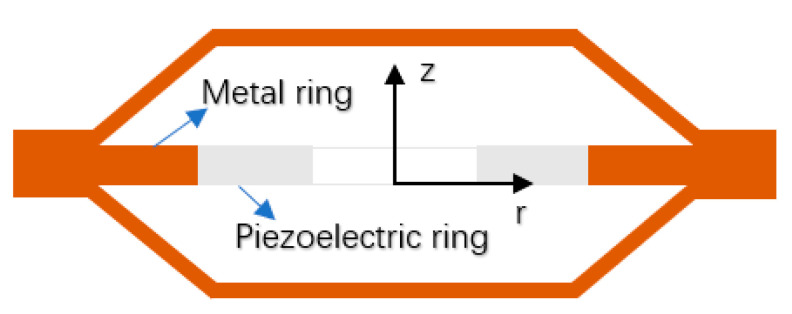
Structure diagram of cymbal with piezoelectric ring and metal ring.

There is a further pressure compensation scheme in the hub cymbal transducer [106,107], as shown in Figure 18. In the hub type design, the tangential stress in the end cap is further reduced, which can improve the energy conversion efficiency and increases the displacement response accordingly. Lin [81,84] proposed to replace the bonding layer by adding a metal ring structure (as shown in Figure 19) to the piezoelectric ceramic sheet.

If cymbal intelligent structure is to be used in underwater applications, especially in deep-water high-pressure areas, its pressure resistance must be studied. According to the research of Erman Uzgur [108], the limit pressure of the intelligent structure is determined by the effective piezoelectric coefficient. As the cavity depth changes, as shown in Figure 13, the limit pressure of intelligent structure increases. And the material properties and the device diameter will have an impact on the ultimate pressure.

**Figure 20:**
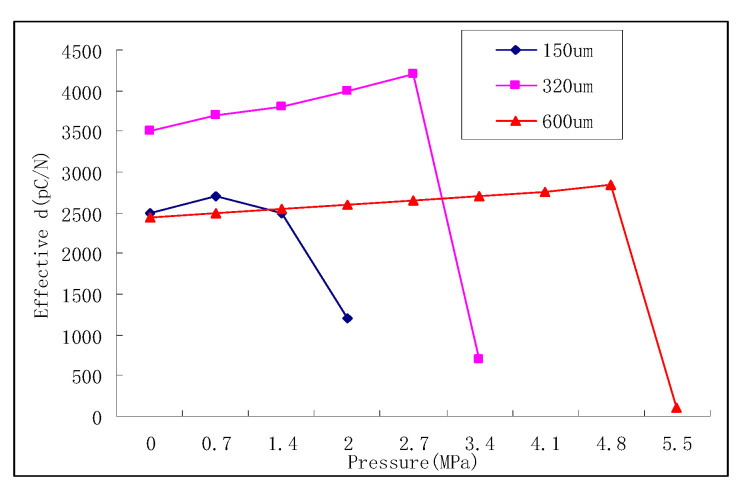
Influence of cavity depth.

**Figure 21:**
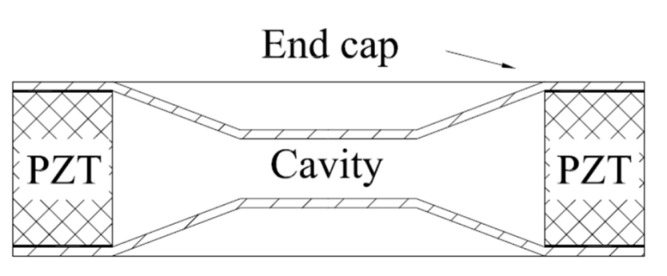
Structure with concave end cap.

In order to meet the pressure requirements in deep-water regimes and broaden the application scope, a piezoelectric ceramic ring is introduced as the core component to improve the pressure resistance of intelligent structures. Zhang [109,110] proposed a cymbal intelligent structure with concave end cap. The pressure resistance of the structure is three times that of cymbal intelligent structure, as shown in Figure 20 and Figure 21. Since the cavity is immersed in liquid, opening holes on the surface of the metal end cap will make the pressure inside and outside the end cap balanced. Therefore, there is no pressure difference to damage the structure.

**Figure 22:**
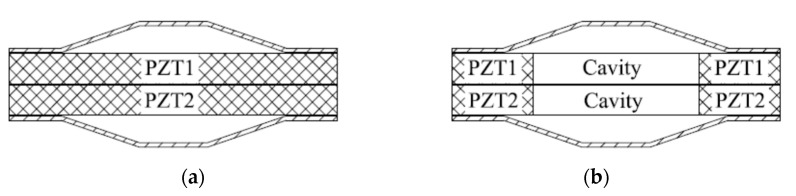
Structure diagrams of dual drive cymbal. (**a**) Two piezoelectric discs (**b**) Two piezoelectric rings.

The dual drive intelligent structure, shown in Figure 22, was first proposed by Zhang [111] in the 21st century. By applying voltage of different size and phase to PZT, the structure has directivity (heart or dipole). Subsequently, a piezoelectric ceramic ring was introduced into it to create a hollow double drive intelligent structure, and patents for this design have been applied for [112,113].

**Figure 23:**
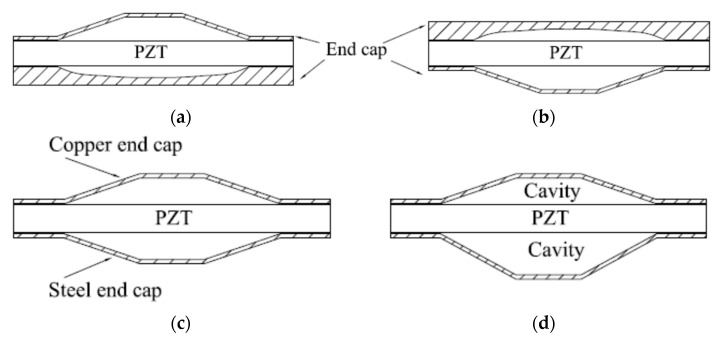
Asymmetric structure diagrams of cymbal. (**a**) Asymmetric configuration of end cap (**b**) Asymmetric configuration of end cap (**c**) Asymmetric material of end cap (**d**) Asymmetric cavity.

In order to broaden the resonance band of intelligent structures, the United States Navy and China proposed an asymmetric intelligent design [114,115]. The asymmetrical properties of two sides of PZT make their resonance bands different, resulting in larger bandwidth. In Figure 23, three types of asymmetric structures developed by the U.S. Navy are shown. The designs feature an asymmetric shape structure of the metal end cap (b), different material of metal end cap (c) and different cavity depth on both sides (d). The first structure (a) originates from China.

**Figure 24:**
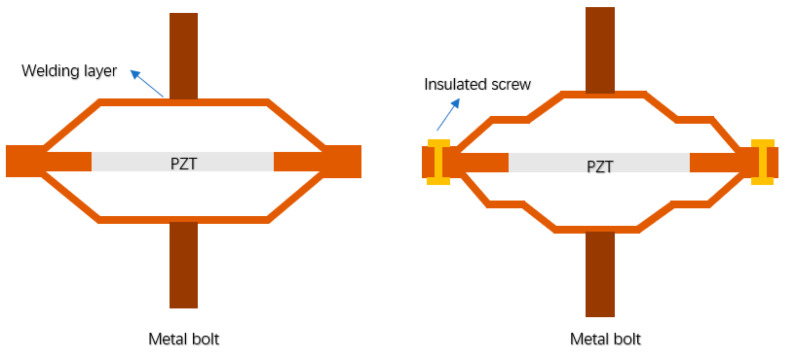
Structure diagram of cymbal with bolt (**left**) and multilayer cavity (**right**).

Choa [116] adds bolts on both sides of the metal end cap, in order to adjust the resonance frequency of the intelligent structure, as shown in Figure 24. The studs can be used as electrodes and increase the mass of metal end caps, so that the resonance frequency of the intelligent structure is reduced. Tang [117,118] applied this structure to an underwater launch device. Wang [119] designed a cymbal transducer with multilayer cavity, which used insulated screws to replace the bond layer in order to increase its intensity. He also used a piezoelectric transducer array in the underwater large target test [120].

**Figure 25:**
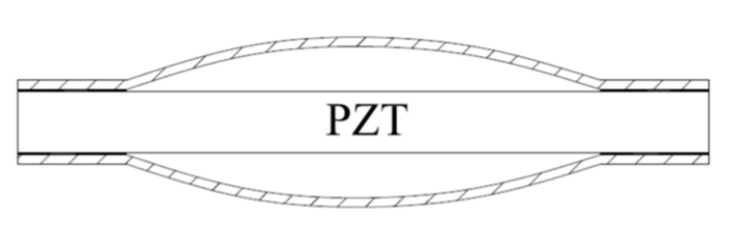
Structure diagram of cymbal with spherical end cap.

Wu [121,122,123,124,125] also proposed the spherical crown intelligent structure shown in Figure 25. Research involving this structure revealed that the spherical end cap can improve the output efficiency and that the design can be applied at increased water depths. Compared with the traditional cymbal transducer, the axial vibration displacement of the spherical crown transducer is increased by 4.55% (in air) and 7.78% (in water) by using a spherical crown metal end cap instead of the traditional cymbal transducer. Meanwhile, the acoustic emission power is increased, and the fundamental resonance frequency is reduced by about 4.41% (in air) and 7.86% (in water), which can be used for acoustic detection in lower frequency bands. The static pressure limit load of the transducer is increased by 9.42% compared with the traditional cymbal transducer.

**Figure 26:**
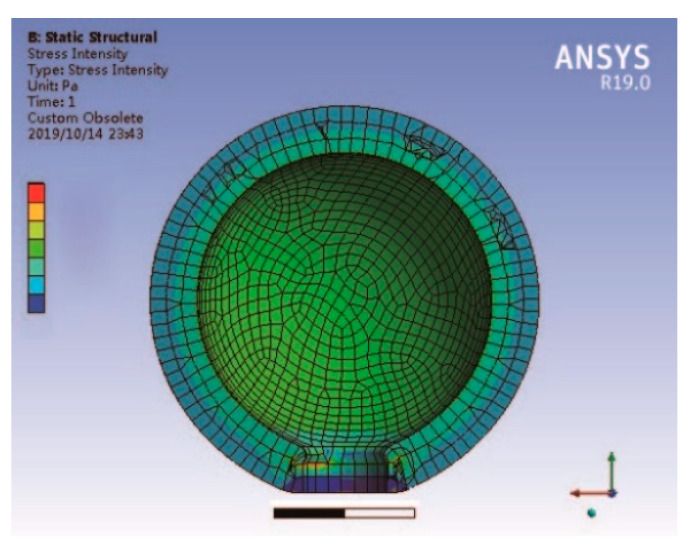
Spherical pressure hydrophone.

Another spherical pressure hydrophone [126], shown in Figure 26, is designed and fabricated by using a radial polarized air backed piezoelectric spherical shell transducer as the acoustic receiving sensitive element. The experimental results show that when the diameter of the spherical pressure hydrophone is 36 mm, the operating frequency is 50 Hz–10 kHz, the low frequency receiving sensitivity is −198.4 dB (0 dB = 1 V/Pa) and the equivalent self-noise spectrum level is 46.5 dB@1 kHz. This designed structure can work at a depth of 3000 m.

**Figure 27:**
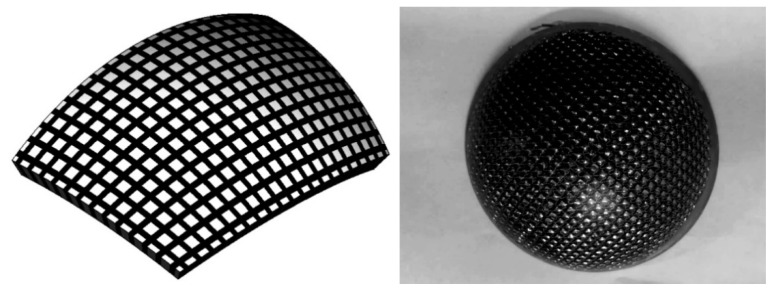
Spherical structure with square piezoelectric ceramics.

Guan [127] proposes a spherical structure with square piezoelectric ceramic material embedded in a spherical polymer surface as shown in Figure 27. This transducer has the advantages of a unique mode in working frequency band and wide beam in high frequency operation, which can be widely used in the development of underwater acoustic detection and transmitted transducer array. This high frequency underwater acoustic transducer based on spherical piezoelectric composite ceramic material has only a single resonant peak in the frequency band of 200–400 kHz. The maximum emission voltage response is 165 dB and the bandwidth of −3 dB is nearly 70 kHz. It has the characteristics of single mode, broadband and wide beam radiation in high frequency operation.

**Figure 28:**
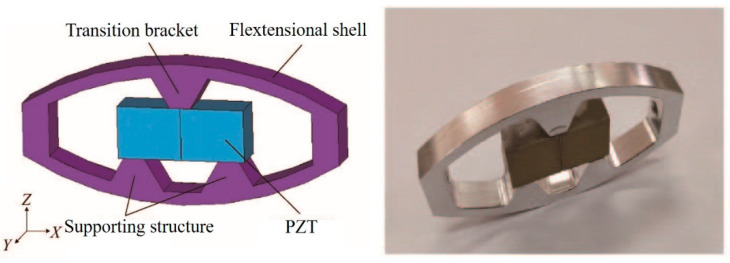
Piezoelectric transducer with transition bracket.

Because of its unique characteristics of shear deformation and high-voltage electrical constant, high electromechanical coupling coefficient and low dielectric constant, the shear vibration mode of piezoelectric ceramics has a good performance in piezoelectric energy harvesters and new structure composite transducers. Through the design of a transition layer with a special structure, the shear vibration of piezoelectric ceramics can be explored. The shear vibration generated by piezoelectric ceramics is transformed into the thickness vibration of composite materials, so as to meet the requirements of underwater acoustic transducer and improve the piezoelectric properties of composite materials. In order to explore the application of piezoelectric shear vibration mode in a flextensional transducer, a new transducer structure [128,129] is proposed in Figure 28. Through the metal bending shell and trapezoidal transition structure, the shear vibration generated by piezoelectric ceramics is transformed into the bending and tensile vibration of metal shell, so as to realize acoustic radiation and increase the acoustic radiation area. When the radiation area of the transducer is 120 mm × 240 mm, the maximum emission voltage response reaches 158.3 dB at the resonance frequency of 101 kHz, and the working bandwidth is 86–114 kHz. The receiving sensitivity is −197 dB and the maximum received signal bandwidth is 48 kHz at −3 dB.

**Figure 29:**
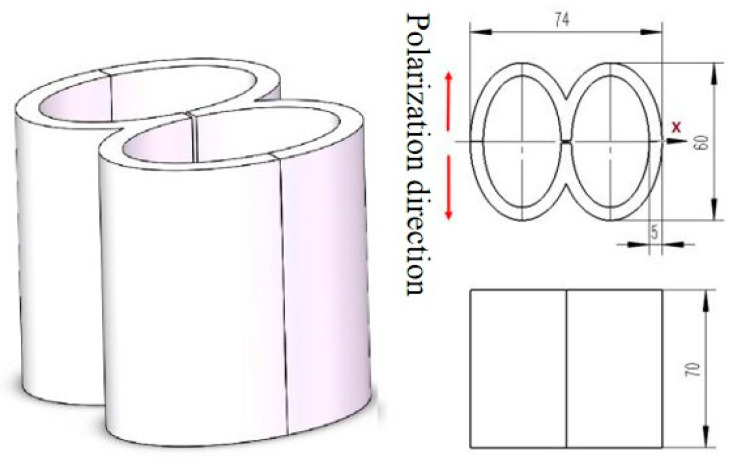
Low frequency transducer with two piezoelectric ceramic elliptical shells.

In addition to the axial transducer, there also exists a radial transducer type. In order to satisfy the demands of small-size and low-frequency a transducer for low frequency active detection of unmanned underwater vehicles has been proposed [130] which is composed of double piezoelectric ceramic elliptical shells. The transducer is used to construct a small low-frequency emission array, and it is mounted on an autonomous underwater vehicle. In Figure 29, the long axis of the elliptical ring is 60 mm in length, the short axis is 40 mm, the height is 70 mm and the thickness is 5 mm. The total weight of the virtual prototype is less than 1.5 kg. The maximum emission voltage response of the transducer at 3 kHz is 130 dB. The maximum linear size of the 16-element array formed by the transducer is 5 m and the maximum emission voltage response of 3.3 kHz is 155 dB. The double piezoelectric ceramic elliptical shell transducer obviously has the characteristics of small size and light weight, which can be applied to underwater vehicles.

**Figure 30:**
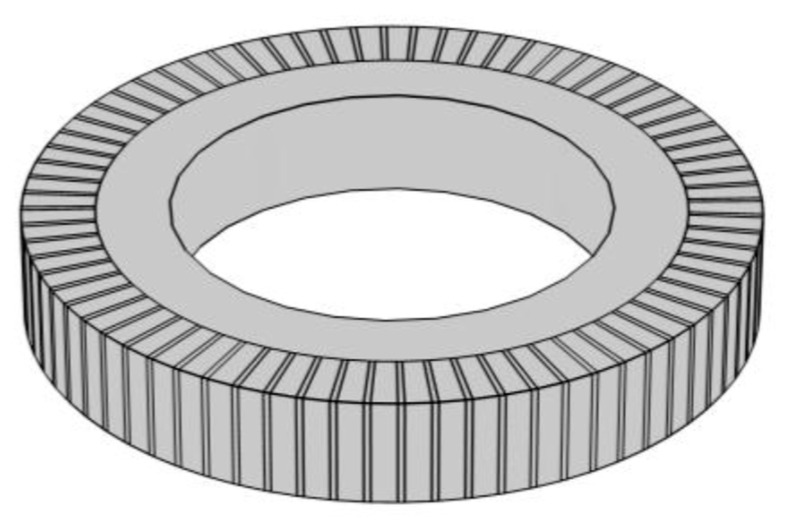
Radial composite piezoelectric ceramic ultrasonic transducer.

Chen [131] designed a new radial composite piezoelectric ceramic ultrasonic transducer with a piezoelectric ceramic thin inner ring and a metal outer ring in Figure 30. The outer ring is composed of 180 equal parts of ceramic elements. The elements are connected by epoxy resin and the material of the inner ring is aluminium. It has a higher electromechanical coupling coefficient, a wider working frequency band and higher sensitivity. The precise transducer mode in the working frequency band can reduce the coupling vibration in other directions as far as possible.

**Figure 31:**
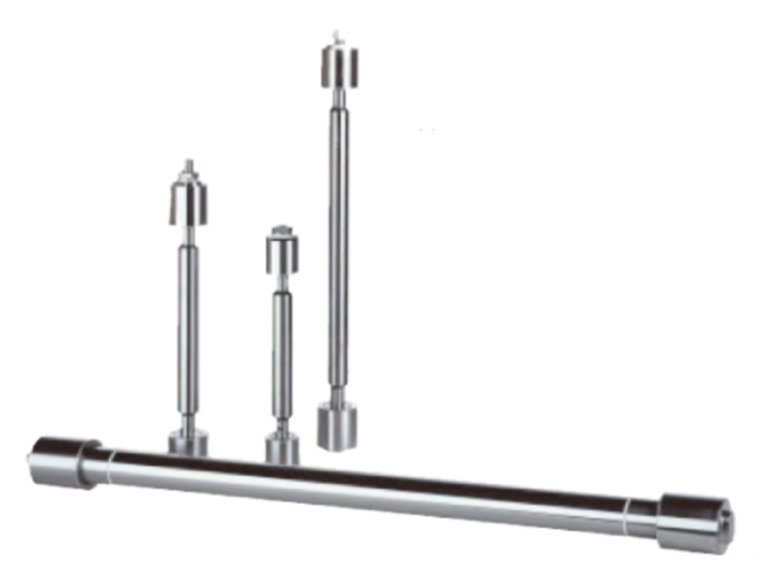
Radial push-pull transducer.

Walter [132] developed the radial push-pull transducer, shown in Figure 31, which can effectively improve the ultrasonic cleaning field. It constitutes a longitudinal vibration composite piezoelectric transducer coupled at both ends (or one end) of a long metal tube to produce an in-phase push-pull effect on the tube, thus generating sound radiation in its radial direction. The power of the single transducer can reach more than 2 kW. The length of the tube is usually an integer multiple of the half wavelength, that is, it works on the integer multiple mode of the fundamental resonance frequency. In fact, the push-pull transducer works in the coupling vibration state rather than a single radial vibration mode. In fact, the circular tube is in a state of standing wave vibration, so the sound field radiated along the tube length is a standing wave field, and the uniformity of the radiated sound field needs to be further improved.

**Figure 32:**
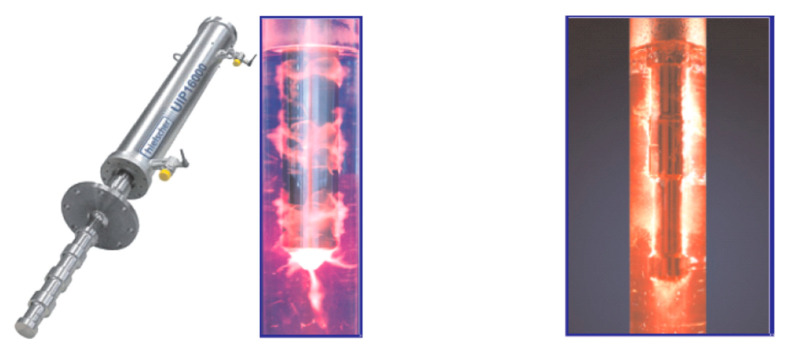
Rod ultrasonic transducer and its better cavitation in water.

The Hielscher company has launched a series of high-power bar ultrasonic transducers [133], as shown in Figure 32. The maximum electric power of this type of transducer can reach 16 kW and it can work under extremely harsh conditions, such as under high temperature and high pressure. The principle is similar to the push-pull transducer. A long metal rod with stepped disk is excited by one or more high-power longitudinal composite piezoelectric ultrasonic transducers, and the length of the rod satisfies the requirement of being an integer multiple of λ/2. The stepped disk is usually designed according to the vibration displacement amplitude or node position of the rod. This increases the effective acoustic radiation area of the rod to improve the acoustic radiation efficiency while it also increases the strength of the round rod. This can prevent the metal rod from fracture resulting from the stress concentration at the displacement amplitude node under high power working condition. In practical application, it is found that, due to the role of the stepped plate, the uniformity of the radiation sound field distribution of rod type ultrasonic transducer in water is better than that of push-pull transducer.

**Figure 33:**
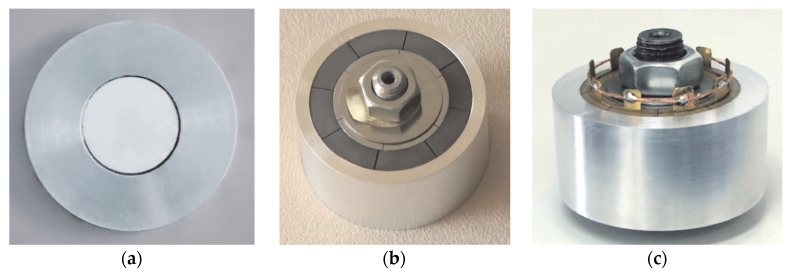
Three radial piezoelectric ultrasonic transducers. (**a**) Radial discs. (**b**) Radial cylinders. (**c**) Sandwiched rings.

The radial composite disk piezoelectric transducer [134] is composed of an inner polarized piezoelectric disk and an outer metal ring, as shown in Figure 33a. In order to improve the power density of the transducer, a metal outer ring is used to exert large radial prestress on the piezoelectric disk. The transducer constitutes a thin disk structure, which can be used as a one-dimensional radial vibration system. The radial vibration frequency equation can be obtained by equivalent circuit theory. This kind of piezoelectric transducer is usually assembled by thermal expansion and cold contraction. In order to ensure uniformity of the transducer performance, high precision interference fit machining and assembly process is required.

Figure 33b displays a three-dimensional radial composite cylindrical piezoelectric ultrasonic transducer [135,136]. The transducer consists of three parts: the outer part is a metal tube, the inner part is a cylindrical metal elastic expansion inner core and the middle layer is a group of the same arc-shaped piezoelectric ceramic pieces, which can be cut equally from the radial polarized piezoelectric ceramic tube. The electric power limit of the transducer can be increased by a certain amount of prestress [137]. The inner part of the transducer constitutes an elastic expansion core with adjustable radial force, which can exert enough radial prestress on the arc-shaped piezoelectric ceramic ring group together with the external metal tube, so as to greatly improve the electric power limit and power density of the transducer.

Another type of three-dimensional composite sandwich radial vibration piezoelectric ultrasonic transducer is shown in Figure 33c [138]. It was developed in recent years, and its structure is similar to that of other radial transducers. The middle layer of the transducer is composed of a group of arc-shaped piezoelectric ceramic rings. Each arc-shaped piezoelectric crystal stack is composed of a number of cylindrical piezoelectric rings along the radial direction. There is a decoupling, facilitated through an air gap, between successive adjacent arc piezoelectric stacks. The two stacked arc-shaped piezoelectric rings are polarized along the radial direction, and their directions of polarization are opposite. The theoretical analysis of its radial vibration characteristics is given in reference.

## 5. Conclusions

We have presented a comprehensive literature review discussing the physical mechanism and structure development of piezoelectric ceramic sandwich structure transducer. Overall, piezoelectric transducers follow the trend of miniaturization, low frequency, high efficiency, and diversification with the continuous optimization of structure and material. Piezoelectric transducers are encountered in a wide range of civil and defence applications. We also compared the advantages and disadvantages of different structures and their scope of application. From the development of the structural evolution, it is not difficult to establish that the structural evolution is manifested in the end cap. Additionally, the core component has gradually developed from its original monolithic structure to composite ring-type structures. In the context of underwater acoustics piezoelectric transducers have been continuously optimized to satisfy the particular requirements of deep water, low frequency and high power. Recent underwater piezoelectric transducers already achieved three-dimensional signal detection instead of the traditional one-way detection. These new transducers feature high electromechanical coupling coefficient, high electromechanical conversion coefficient and high-quality characteristics. Moreover, piezoelectric ultrasonic transducers with both circumferential and axial structures can work with high power in extremely harsh environments such as in high temperature and under high pressure.

Future developments may focus on the three-dimensional or spatialized design of piezoelectric resonators, especially in the field of underwater acoustics. Miniaturization, intelligence and high efficiency will, naturally, always remain the focus of research. It is believed that piezoelectric transducers will continue to innovate and make far reaching contributions in the future life to improve human life and production.

## Figures and Tables

**Table 1:** Types, applications and characteristics of different piezoelectric ceramics.

Ceramic Type	Material Name	Applications	Characteristics
Soft PZT ceramic	PZT-51	low-power ultrasonic transducers	large piezoelectric constants; high permittivity, large dielectric constants, high dielectric losses, large electromechanical coupling factors, low mechanical quality factors, a low coercive field, poor linearity, easy to depolarize.
	PZT-52	low-frequency sound transducers	
	PZT-53	applications with high coefficient	
	PZT-5H	microphones, vibration pickups with preamplifier	
	PLiS-51	low-frequency vibration measurements	
	PMgN-51	Hydrophones, transducers in medical diagnostics	
	PSnN-5	Actuators	
Hard PZT ceramic	PZT-41		small piezoelectric constants, low permittivity, small dielectric constants, low dielectric losses, small electromechanical coupling factors, high mechanical quality factors, high coercive field, good linearity, hard to depolarize.
	PZT-42	High-power acoustic applications	
	PZT-43	Hydroacoustics, sonar technology	
	PZT-82	piezomotor	
	PCrN-4		
	PBaS-4		
Lead free Piezo Ceramic	BaTiO_3_	Ultrasonic transducers suitable for low-temperature underwater, for example Ultrasonic Transducer in fishfinder	Low density, low curie temperature, lead free.

**Table 2:** Performance parameters of piezoelectric materials.

Material		P-41	P-51	P-81	PbaS-5	BaTiO_3_	PZT-5X
Elastic compliant constant	*S*_11_ (10^−12^ m^2^/N)	12	16.7	11.1	13.5	8.4	19
Dielectric constant	*ε*	1050	2200	1000	1650	1550	4500
Dielectric loss	tg(%)	<0.3	2	0.5	0.5	0.5	2
Quality factor	*Q_m_*	1000	80	800	1800	1300	65
Electromechanical coupling coefficient	*k_p_*	0.58	0.62	0.52	0.59	0.34	0.7
	*k*_31_	0.34	0.35	0.3	0.34	0.196	0.4
	*k*_33_	0.66	0.69	0.6	0.6	0.43	0.77
	*k_t_*	0.48	0.5	0.45	0.47	0.32	0.53
Piezoelectric constants	*d*_31_ (−10^−12^ C/N)	113	186	90	150	150	300
	*d*_33_ (10^−12^ C/N)	260	600	220	330	330	750
	*g*_31_ (10^−3^ Vm/N)	12	10	11.2	10	10	8
	*g*_33_ (10^−3^ Vm/N)	28	34	24.8	22	22	17.5

## Data Availability

The data presented in this study are available on request from the corresponding author.
